# FACTORS ASSOCIATED WITH A FOUR-YEAR SARCOPENIA TRANSITION IN CHINESE OLDER ADULTS

**DOI:** 10.1093/geroni/igad104.3801

**Published:** 2023-12-21

**Authors:** Shujing Lin, Lingxiao He, Jinzhu Yang, Ya Fang

**Affiliations:** Xiamen University, Xiamen, Fujian, China (People’s Republic); Xiamen University, Xiamen, Fujian, China (People’s Republic); Xiamen University, Xiamen, Fujian, China (People’s Republic); Xiamen University, Xiamen, Fujian, China (People’s Republic)

## Abstract

**Background:**

Previous studies have reported a dynamic change of sarcopenia status while factors associated with such status changes remained unexplored. This study aimed to identify factors associated with sarcopenia status transitions based on Chinese older adults.

**Methods:**

6065 older adults (aged 66.8±6.2) with complete data at baseline and at least one follow-up (2 years) were included from the China Health and Retirement Longitudinal Study (CHARLS). Sarcopenia was determined based on AWGS2019 criteria. Multistage Markov model was used to explore potential sociodemographic, behavioral, psychological and disease-related factors associated with sarcopenia transitions.

**Results:**

At baseline, 59.1% of participants were non-sarcopenic, 24.7% had probable sarcopenia and 16.2% were diagnosed with sarcopenia. Participants with non-probable sarcopenia had 1-year transition probability of developing probable sarcopenia (0.101) or sarcopenia (0.056). Overweight (HR=1.89, 95%CI: 1.55-2.30), obesity (HR=1.85, 95%CI:1.37-2.48) and presence of chronic diseases (HR=1.26, 95%CI: 1.03-1.56) increased the risk of progression from non-sarcopenia to probable sarcopenia. Older age (HR=1.10, 95%CI:1.08-1.13), female sex (HR=1.38, 95%CI: 1.08-1.75), shorter sleep duration (HR=1.42, 95%CI:1.12-1.80), depression (HR=1.27, 95%CI:1.01-1.59) and presence of chronic diseases (HR=1.35, 95%CI: 1.05-1.74) were associated with transition towards sarcopenia from non-sarcopenia. Higher cognitive function (HR=1.03, 95CI:1.01-1.05), rural dwelling(HR=1.25, 95%CI: 1.00-1.55) and alcohol consumption (HR=1.41, 95%CI:1.15-1.73) were associated with reversed status from probable sarcopenia to non-sarcopenia. Higher cognitive function increased the conversion from sarcopenia to probable sarcopenia (HR=1.21, 95%CI:1.06-1.37) and non-sarcopenia (HR=1.03, 95%CI:1.00-1.06) .

**Conclusions:**

Sarcopenia is a dynamic condition in older adults. Possible interventions should focus on improving cognitive functions, sleep quality and better controlling on chronic conditions.

